# Effects of Anti-TNF Alpha Drugs on Disability in Patients with Rheumatoid Arthritis: Long-Term Real-Life Data from the Lorhen Registry

**DOI:** 10.1155/2014/416892

**Published:** 2014-07-06

**Authors:** Matteo Filippini, Chiara Bazzani, Fabiola Atzeni, Piercarlo Sarzi Puttini, Antonio Marchesoni, Ennio Giulio Favalli, Roberto Caporali, Lorenzo Cavagna, Roberto Gorla

**Affiliations:** ^1^Rheumatology and Immunology Unit, Spedali Civili di Brescia, Piazza le Spedali Civili 1, 125123 Brescia, Italy; ^2^Rheumatology Unit, L. Sacco University Hospital, Via G.B.Grassi 74, 20157 Milan, Italy; ^3^Rheumatology Day Hospital, G. Pini Orthopedic Institute, Milan University, Piazza Ferraari, 20122 Milan, Italy; ^4^Rheumatology Division, University and IRCCS Policlinico S. Matteo Foundation, Piazzale Golgi 2, 27100 Pavia, Italy

## Abstract

This study involving 1033 patients with RA confirms the effectiveness of etanercept, adalimumab, and infliximab in reducing RA-related disability even in patients with a history of highly active and longstanding RA. Moreover, we found that the improvement in disability was biphasic, with a marked improvement during the first year of anti-TNF therapy, followed by slower but significant recovery over the subsequent four years.

## 1. Introduction

Rheumatoid arthritis- (RA-) related disability is one of the major problems faced by clinicians and patients: it reduces working capacity [1], affects the personal relationships and lifestyles of patients and their relatives [2], and increases the direct and indirect costs of the disease [3]. The wide range of factors that may give rise to patient disability include disease activity, joint damage [4], articular pain [5], and comorbidities [6, 7]. However, despite the established impact of disability in RA, the current treatment guidelines are driven by evaluations of disease activity based on composite scores such as the 28-joint disease activity score (DAS28). Introduced in 1995 [8], the DAS28 has a cut-off value of <2.6 defining RA remission [9] but does not include a disability assessment. Moreover, real-life practice clearly shows that multiple joints can remain swollen or tender, and that pain can persist even when patients meet the remission cut-off score [10]. It is interesting to note that a recent large-scale observational study found disparities between the reduction in disease activity as expressed by DAS28 scores and the progression of disability [11]. The recently published ACR/EULAR remission criteria are also affected by these limitations [12]. The fact that the available disease activity scores do not necessarily correlate with structural remission or disability therefore suggests that there is a need for additional means of evaluation and a more detailed consideration of the quality of remission [13].

This is particularly important because the therapeutic approach to RA has greatly improved as a result of its earlier diagnosis and treatment [14, 15] and the availability of bio(techno)logical drugs such as anti-TNF*α* agents [16]. The European League Against Rheumatism (EULAR) recommendations stress the well-timed use of anti-TNF agents in the case of the premature failure of traditional disease modifying antirheumatic drugs (DMARDs) [17].

The Health Assessment Questionnaire (HAQ) is the most widely used index of disability in RA: it is sensitive, effective, reliable, cheap and rapid to administer, reflects the patients' point of view, and correlates well with measures of chronic inflammation [18]. If an HAQ score is <0.5 during a year, RA treatment can be considered very effective, but this is true of only 38% of the patients with a DAS28 score of <2.6, and 56% of those with the HAQ a simple disease activity index (SDAI) of <3.3 [18]. In addition, HAQ is related to working capacity [19], the need for specialist examinations [20], and the* quoad vitam* prognosis [21], and is also an appropriate means of summarising outcomes and the direct and indirect costs of the disease [22].

The primary aims of this study were to define the long-term effects of anti-TNF*α* drugs (etanercept, adalimumab, and infliximab) on disability in patients with early or long-standing RA and evaluate whether an improvement in HAQ scores correlates with an improvement in DAS28 scores. The secondary aims included identifying the baseline factors associated with disability, evaluating the kinetics of drug-induced improvements in disability, and indirectly observing whether there are differences in functional responses to the three anti-TNF drugs.

## 2. Materials and Methods

The source of the data used in this study was the online Lombardy Rheumatology Network (LORHEN) registry, which contains the clinical history and demographic data of all patients satisfying the 1987 revised American College of Rheumatology (ACR) criteria for RA [23] attending four Rheumatology Centres in Lombardy (Spedali Civili in Brescia, Ospedale L. Sacco and Istituto G. Pini in Milan, and Policlinico San Matteo in Pavia) since 1999 who have been treated with bio(techno)logical drugs until last year. The registry has been previously used as a source for other scientific publications [24, 25]. The inclusion criteria were beginning first-line bio(techno)logical treatment with an anti-TNF agent (infliximab, adalimumab, or etanercept) and at least six months of followup. The data were collected at baseline and then every six months until a maximum followup of 60 months (end of collection: March 2013) and included the number of swollen and tender joints (out of 28 joints), laboratory findings (rheumatoid factor (RF), anticitrullinated protein antibodies (ACPAs), C-reactive protein (CRP) levels, the erythrocyte sedimentation rate (ESR)), and DAS28 and HAQ scores [26].

The enrolled patients were stratified on the basis of different variables: age at the time of beginning anti-TNF*α* therapy (≥65 versus <65 years); gender (males versus females); RF (seronegative versus <3 times the upper normal limit of 42 IU/mL (low titre) versus ≥3 times the upper normal limit (medium/high titre)); disease duration at baseline (<3, 3–5, 5–10, 10–15, or ≥15 years); disease activity at baseline assessed on the basis of DAS28 scores (<2.6 = remission; 2.6–3.2 = low disease activity; 3.3–5.0 = moderate disease activity; ≥5.1 = high disease activity); baseline HAQ scores (<0.5 = no disability; 0.5–1 = mild disability; 1-2 = moderate disability; ≥2 = severe disability) [27]; the concurrent use of DMARDs and steroids (yes versus no); the anti-TNF agents used (infliximab, etanercept, and adalimumab); and the number of comorbidities (1, 2, 3, or ≥4), including all comorbidities that have a potential impact on patient disability such as cardiovascular and lung involvement, peripheral neuropathy, type 2 diabetes mellitus, dyslipidemia, thyroid illness, and osteoporosis.

The improvement in disability was considered clinically significant if it was more than the minimally important difference (MID; HAQ > 0.22) [28].

## 3. Statistical Analysis

The differences between the anti-TNF agents were analysed on the basis of the data related to all LORHEN patients with at least a 6-month HAQ score using the Kruskal-Wallis nonparametric test for continuous variables (mean values and standard deviations) and the chi-squared test for categorical variables (absolute numbers and percentages). The changes from baseline were analysed using Wilcoxon's signed-rank test. The multivariate analyses were made using stepwise logistic regression models, with the response variable being defined as a >0.5 decrease in HAQ scores after one and five years. All of the analyses were made using SAS version 9.2 (SAS Institute, Inc., Cary, NC), and a *P* value of 0.05 or less was considered statistically significant.

All of the statistical analyses excluded patients with missing data.

## 4. Results

The LORHEN registry includes 1381 patients satisfying the 1987 revised ACR criteria for RA [24]. We considered only those receiving infliximab, adalimumab, or etanercept as a first-line bio(techno)logical drug.

The final study population consisted of 1033 patients (847 females, 186 males) whose baseline clinical and demographic characteristics are shown in Tables 1 and 2. At the end of the study, 42% of the patients were still receiving an anti-TNF*α* agent (Figure 3). Disability as assessed on the basis of their HAQ scores significantly decreased in all cases (ΔHAQ −0.78; *P* < 0.05): at the end of the followup, the HAQ score was 0.69 (mild disability) as against 1.42 at baseline (moderate disability). Furthermore, 193/904 patients (21.35%) had no disability (HAQ < 0.5) after one year and 123/344 (35.75%) after five years of followup.

**Figure 1:**
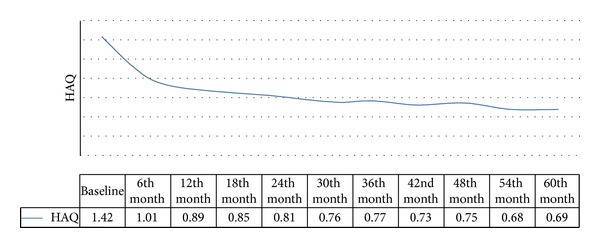


The change in HAQ scores over time had a biphasic course (Figure 1), with a rapid clinical improvement in the first year (ΔHAQ 0-1: −0.53; *P* < 0.05), followed by a further slower improvement that did not become clinically or statistically significant until the end of the followup (ΔHAQ 1–5: −0.25; *P* < 0.05). The functional improvement was even more striking (ΔHAQ 0–5: −0.81; *P* < 0.05) among the 459 patients in clinical remission (44.43%; DAS28 < 2.6), and there was no disability after the 30th month of followup. Interestingly, there was a temporal dissociation between the time of clinical remission (21st month) and the time of maximum functional improvement (60th month).

The population was then stratified on the basis of the various clinical and demographic variables described in the Methods section, and the results are shown in Table 3. The 847 females (82%) had greater disability at baseline than the 186 males (18%) (ΔHAQ > MID; *P* < 0.05), and this difference was even greater at the end of the followup period.

The 245 patients aged ≥65 years (25.76%) showed greater disability at baseline (ΔHAQ > MID; *P* < 0.05) than the 788 patients aged 18–64 years (74.24%), and their improvement during followup was less striking.

The seronegative patients (75/668, 11.22%) and those with a low RF titre (110/668, 16.47%) showed similar disability at baseline and similarly improved during the five years of biological treatment (HAQ < MID; *P*: ns), whereas the baseline disability of the patients with a high titre (483/668, 72.31%) was worse (ΔHAQ > MID; *P* < 0.05) and remained so until the end of the followup.

The between-group differences in disease duration before the start of anti-TNF therapy (<3 years: 412/1017, 40.52%; 3–5 years: 105/1017, 10.32%; 5–10 years: 181/1017, 17.8%; 10–15 years: 140/1017, 13.76%; and >15 years: 179/1017, 17.6%) were significant at baseline and remained so until the end of the followup (ΔHAQ > MID; *P* < 0.05): in particular the longer the disease duration, the worse the disability.

The patients with higher baseline HAQ and DAS28 scores showed worse disability and achieved a less significant improvement (ΔHAQ > MID; *P* < 0.05). The disability subgroups were HAQ < 0.5: 53/1018, 5.2%; 0.5–1: 161/1018, 15.82%; 1-2: 599/1018, 58.85%; and ≥2: 205/1018, 20.13%. The disease activity subgroups were DAS28 < 3.2: 30/1021, 2.93%; 3.2–5.1: 218/1021, 21.36%; ≥5.1: 773/1021, 75.71%.

The patients were stratified into four subgroups on the basis of the number of comorbidities: 380/704 (53.98%) had one, 188/704 (26.7%) two, 86/704 (12.22%) three, and 50/704 (7.1%) four or more. The presence of comorbidities significantly reduced the recovery of joint function during anti-TNF treatment: the greater the number of comorbidities, the worse the improvement in disability (ΔHAQ > MID; *P* < 0.05).

The baseline difference between the patients receiving steroids (867/1033, 83.93%) or not (166/1033, 16.07%) was statistically significant (ΔHAQ > MID; *P* < 0.05), but there was no difference after 42 months (ΔHAQ > MID; *P* = ns).

The patients concurrently receiving DMARDs (950/1033, 91.94%) showed a better functional recovery than those receiving monotherapy (83/1033, 8.03%), and the difference became significant after 30 months of followup (ΔHAQ > MID; *P* < 0.05).

In relation to the anti-TNF*α* agents used (infliximab, 494/1022, 48.34%; etanercept 227/1022, 22.21%; and adalimumab 301/1022, 29.45%), the patients treated with infliximab had worse baseline disability (ΔHAQ > MID; *P* < 0.05), but the difference became nonsignificant after two years of anti-TNF treatment (ΔHAQ > MID; *P* = ns).

**Figure 2:**
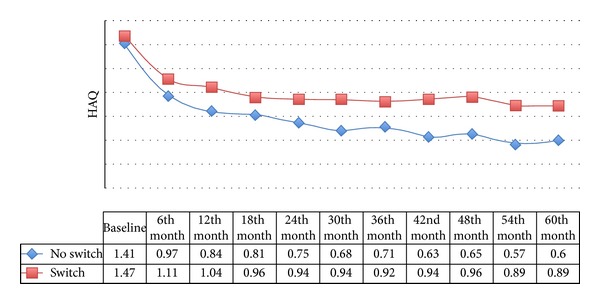


**Figure 3:**
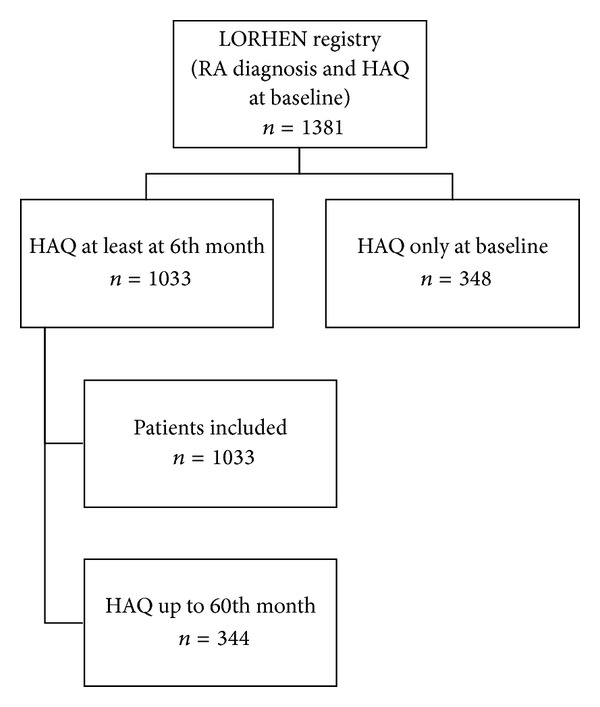


Two hundred and seventy-five patients (26.62%) switched from the first to a second TNF blocker because of a secondary lack of effectiveness (58.55%), adverse events (30.55%), or other reasons (10.9%): the most frequently discontinued drug was infliximab, which was most frequently replaced by etanercept. The patients who did not need to modify their biological treatment had less residual disability than those who had to switch (HAQ 0.6 versus 0.89; *P* < 0.05) (Figure 2), but the improvement in HAQ scores was significant after five years of followup in both subgroups (ΔHAQ > MID; *P* < 0.05).

Multivariate analyses identified the baseline variables correlated with a more than 0.5 decrease in HAQ scores after one and five years. The patients who were less likely to achieve an optimal functional recovery were mainly females, aged more than 65 years, had a baseline DAS28 score of >5.1, and were not taking steroids (Table 4). Functional recovery after five years was reduced in the patients treated with etanercept or adalimumab.

It was impossible to include the other variables in the multivariate analyses because of the missing data.

## 5. Discussion

Our results confirm the effectiveness of etanercept, adalimumab, and infliximab in reducing RA-related disability even in patients with a history of highly active and long-standing RA. Similar findings have been reported in other, shorter observational studies [29, 30]. As suggested by Aletaha and Ward, we can also confirm that patients can achieve a good functional recovery even after years of illness because the reversible factors underlying HAQ scores (pain and inflammation) tend to prevail over joint damage [31]. Starting anti-TNF therapy not only generally reduced disability from moderate to mild, but also led to patients who achieved clinical remission during the followup (DAS28 score <2.6) completely recovering from disability (HAQ score <0.5) regardless of disease duration. Randomised clinical trials have shown that the well-timed use of biological treatment in the case of early failure with traditional DMARDs should induce the complete remission of functional dysfunction [32, 33], but these findings require confirmation in further studies aimed at this primary endpoint.

We found that the improvement in disability was biphasic, with a marked improvement during the first year of anti-TNF therapy, followed by slower but significant recovery over the subsequent four years. This was typically observed when a “step-up” therapeutic strategy was adopted and, in this case, it is important to wait five years for the stabilisation of HAQ scores in patients with long-standing RA. More aggressive and earlier treatment leads to a rapid phase of improvement (a J-shaped curve) and subsequent stabilisation after one year [24]. However, it is not enough to consider a state of clinical remission defined on the basis of DAS criteria because, in our study, DAS28 remission was observed an average of 21 months after the start of anti-TNF treatment, whereas it took five years before the improvement in HAQ scores was at its peak.

It is interesting to note that there was a clinical and statistical improvement in HAQ scores in all of the analysed groups during followup, although the results were less striking in patients aged ≥65 years, females, and those with a disease duration of more than 10 years, a higher comorbidity index, greater disease activity and disability, a high RF titre, or contraindications to combination therapy with traditional DMARDs. The chronic use of low-dose steroids seemed to contribute to reducing disability in the patients with greater impairment at baseline.

As expected, the patients treated with infliximab (the first biological treatment available in Italy) showed greater disability at baseline, but their improvement was better than that of the patients treated with etanercept or adalimumab.

The univariate and multivariate analyses confirmed that age (≥65 years), female gender, a DAS28 score of >5.1, and no use of steroids correlated with a lower level of recovery from disability during anti-TNF therapy. Although the probability of drug withdrawal was greater with infliximab, the use of etanercept and adalimumab was also associated with a reduced probability of functional recovery, but this was only evident after five years of treatment. Disease duration does not seem to be a negative predictor.

One final aspect emerging from our study concerns switching from one anti-TNF agent to another: the results in the patients who had to switch because of inefficacy or adverse events were less striking than those in the patients who continued on the same drug. These findings are similar to those of other observational studies [16], but the effect on disability of using a drug with a different mechanism of action from the first still remains to be explored.

Recent studies have stressed the importance of measuring functional ability and considering it when making decisions concerning patient management. A Canadian observational study of 1086 RA patients (799 treated with anti-TNF agents) found that the annual mean (direct and indirect) costs of the disease were directly proportional to disability as measured by means of the HAQ: the costs were three times higher for patients with severe disability (HAQ > 2) than for patients without disability (HAQ < 0.5), with working disability being the major indirect cost [34]. Data from the British registry indicates that half of all biological agent-naïve RA patients are unable to work: HAQ scores closely correlated with working capacity, with patients who were severely disabled at baseline being more likely to become work disabled at followup [35].

The main strengths of our study are the large number and the heterogeneity of the enrolled patients, and the long-term followup. However, it also has some important limitations. First of all, as it was an observational study, there may be some patient selection bias. Secondly, RA disability was evaluated without using radiological parameters, and some other parameters (cigarette smoking, ACPAs) were not statistically analysed because there were too many missing data.

## 6. Conclusions

The effect of anti-TNF therapy on the disability of patients with RA is certainly substantial also in the case of long-standing disease and, in addition to other clinimetric indices such as the DAS28, HAQ scores are a good means of evaluating the efficacy of biological treatment.

## Figures and Tables

**Table 1:** Baseline clinical data of the 1033 study patients.

HAQ (SD)		1.42 ± 0.61
DAS28 (SD)		5.73 ± 1.15
	High	773 (74.83%)
	Moderate	218 (21.1%)
	Low	15 (1.45%)
	Remission	15 (1.45%)
	Missing	12 (1.16%)
Steinbrocker functional class	I	37 (3.58%)
	II	706 (68.34%)
	III	247 (23.91%)
	IV	37 (3.58%)
	Missing	6 (0.58%)
Pain visual analogue scale (SD)		62.09 ± 22.68
Global health assessment (SD)		59.81 ± 22.47
Swollen joint count (SD)		9.73 ± 5.54
Tender joint count (SD)		10.8 ± 6.49
Anti-TNF drugs	Adalimumab	305 (29.53%)
	Etanercept	231 (22.36%)
	Infliximab	497 (48.11%)
Number of previous DMARDs	1	67 (6.49%)
	2	364 (35.24%)
	3	309 (29.91%)
	4	172 (16.65%)
	≥5	113 (10.94%)

HAQ: Health Assessment Questionnaire; DAS28: 28-joint disease activity score; TNF: tumour necrosis factor; DMARDS: disease modifying antirheumatic drugs; SD: standard deviation.

**Table 2:** Baseline demographic data.

F/M	847/186 (81.99%)
Age at diagnosis (SD)	46.79 ± 14.62
Age at anti-TNF start, years (SD)	55.12 ± 13.4
Disease duration, years (SD)	7.68 ± 8.15
Comorbidities	704/1033 (68.15%)

F: female; M: male; SD: standard deviation.

**Table 3:** Patients stratified by their clinical and demographic characteristics. The table shows the number of patients in each subgroup and their mean HAQ scores (except in the case of missing data). The bold indicate that the difference between the subgroups is statistically significant (*P* < 0.05).

		Baseline	6th month	12th month	18th month	24th month	30th month	36th month	42th month	48th month	54th month	60th month
Gender	F; M	847-186	847-186	741-163	660-145	603-136	413-82	529-112	374-81	401-78	303-62	281-63
	HAQ	**1.47-1.21**	**1.06-0.75**	**0-0.96-0.59**	**0.91-0.56**	**0.87-0.54**	**0.8-0.55**	**0.82-0.5**	**0.8-0.44**	**0.81-0.42**	**0.73-0.43**	**0.76-0.41**

Age	<65; ≥65; missing	706-245-82	706-245-82	617-218-69	554-193-58	518-172-49	375-106-14	463-142-36	344-97-14	362-107-10	269-84-12	260-76-8
	HAQ	**1.39-1.62**	**0.94-1.21**	**0.81-1.16**	**0.75-1.15**	**0.72-1.09**	**0.68-1.05**	**0.68-1.08**	**0.64-1.04**	**0.67-1.01**	**0.59-0.96**	**0.61-0.98**

RF	neg; <3x; ≥3x; missing	75-110-483-365	75-110-483-365	63-104-416-321	55-95-371-284	52-91-327-269	32-57-181-225	47-84-276-234	34-51-165-205	29-56-198-196	24-43-138-160	22-39-139-144
	HAQ	**1.19-1.2-1.31**	0.82-0.89-0.96	**0.76-0.75-0.88**	**0.71-0.73-0.84**	**0.7-0.7-0.82**	**0.55-0.62-0.75**	0.62-0.68-0.79	0.62-0.56-0.76	**0.58-0.56-0.78**	0.56-0.49-0.72	0.64-0.54-0.71

Disease duration (years)	<3; 3-5;5-10; 10-15; ≥15	412-105-181-140-179-16	412-105-181-140-179-16	367-94-150-122-158-13	331-77-131-114-139-13	294-69-120-105-138-13	190-50-88-69-89-9	249-62-108-94-115-13	170-45-76-69-85-10	175-44-81-74-96-9	140-30-59-54-73-9	128-28-52-55-74-7
	HAQ	**1.23-1.36-1.44-1.64-1.74**	**0.96-0.8-0.86-1.12-1.3**	**0.8-0.75-0.76-1-1.24**	**0.75-0.72-0.68-1-1.21**	**0.69-0.69-0.72-0.89-1.13**	**0.63-0.68-0.67-0.85-1.11**	**0.64-0.67-0.67-0.9-1.08**	**0.65-0.65-0.59-0.78-1.03**	**0.62-0.71-0.66-0.86-0.99**	0.6-0.68-0.62-0.7-0.86	**0.56-0.61-0.58-0.83-0.93**

Comorbidity	1; 2; 3; ≥4; missing	380-188-86-50-329	380-188-86-50-329	335-163-78-45-283	305-146-67-41-246	284-128-62-38-227	190-92-42-30-141	249-114-50-34-194	166-94-39-29-127	177-98-44-27-133	131-80-34-20-100	129-73-32-20-90
	HAQ	**1.42-1.53-1.64-1.68**	**1-1.14-1.28-1.08**	**0.84-1.04-1.2-1.18**	**0.81-0.95-1.16-1.19**	**0.8-0.85-1.16-1.01**	**0.7-0.86-1.24-1.23**	**0.76-0.83-1-0.99**	**0.69-0.76-1.21-1.08**	**0.77-0.75-1.07-1.11**	**0.66-0.7-0.92-1.2**	**0.65-0.84-0.86-1.14**

Baseline HAQ score	<0.5; 0.5-1; 1-2; ≥2; missing	53-161-599-205-15	53-161-599-205-15	46-139-532-174-13	40-118-490-143-14	38-108-444-135-14	23-65-310-88-9	34-86-389-118-14	25-50-293-77-10	25-58-286-98-12	21-40-231-64-9	21-37-208-69-9
	HAQ	**0.48-0.79-1.37-2.35**	**0.3-0.56-0.99-1.61**	**0.31-0.5-0.85-1.52**	**0.25-0.5-0.81-1.44**	**0.29-0.47-0.77-1.35**	**0.14-0.46-0.71-1.33**	**0.15-0.5-0.71-1.32**	**0.14-0.47-0.71-1.17**	**0.15-0.43-0.69-1.23**	**0.14-0.51-0.64-1.1**	**0.14-0.43-0.63-1.15**

Baseline DAS28	<3.2; 3.2-5.1; ≥5.1; missing	30-218-773-12	30-218-773-12	29-194-671-10	26-161-607-11	21-160-547-11	11-105-370-9	18-135-477-11	12-98-335-10	17-99-354-9	13-73-272-7	15-65-258-6
	HAQ	**1.01-1.12-1.54**	**0.55-0.78-1.1**	**0.59-0.69-0.97**	**0.72-0.63-0.92**	**0.33-0.63-0.88**	**0.31-0.55-0.84**	**0.27-0.57-0.84**	**0.38-0.56-0.8**	**0.39-0.64-0.8**	**0.37-0.52-0.74**	**0.35-0.61-0.74**

Anti-TNF drug	ADA; ETA; INF; missing	301-227-494-11	301-227-494-11	260-193-442-9	220-171-404-10	208-153-368-10	159-97-231-8	183-128-320-10	143-84-219-9	133-84-254-8	102-68-188-7	80-57-201-6
	HAQ	**1.24-1.37-1.57**	**0.86-0.99-1.1**	**0.77-0.89-0.97**	0.76-0.88-0.89	**0.7-0.87-0.84**	0.73-0.84-0.75	0.7-0.86-0.77	0.67-0.86-0.72	0.65-0.92-0.74	0.67-0.74-0.66	0.72-0.71-0.67

Steroids	No; yes	166-867	166-867	148-756	132-673	124-615	87-408	107-534	83-372	85-394	68-297	65-279
	HAQ	**1.29-1.45**	**0.88-1.03**	**0.74-0.92**	**0.72-0.87**	**0.68-0.83**	**0.65-0.78**	**0.65-0.79**	0.67-0.75	**0.65-0.77**	0.64-0.69	0.61-0.71

DMARDs	No; yes	83-950	83-950	68-836	53-752	51-688	38-457	48-593	36-419	38-441	29-336	25-319
	HAQ	1.53-1.42	1.05-1	0.97-0.89	0.91-0.84	0.9-0.8	**1.11-0.73**	0.85-0.76	**0.89-0.72**	**0.97-0.73**	**0.9-0.66**	**1-0.67**

F: female; M: male; RF: rheumatoid factor; <3x RF: low titre [<3 times the upper normal limit of 42 IU/mL]; HAQ: Health Assessment Questionnaire; DAS28: disease activity score in 28 joints; TNF: tumour necrosis factor; ADA: adalimumab; ETA: etanercept; INFL: infliximab; DMARDs: disease modifying antirheumatic drugs.

**Table 4:** Univariate and multivariate analyses of baseline characteristics predicting complete disability recovery (HAQ < 0.5).

HAQ < 0.5 (1st year)	Univariate analysis		HAQ < 0.5 (1st year)	Multivariate analysis	
	OR (95% CI)	*P*		OR (95% CI)	*P*
Age (≥65 versus <65 years)	[0.974 (0.962–0.985)]	<0.0001	Age (≥65 versus <65 years)	[0.978 (0.965–0.99)]	0.0004
Gender (F versus M)	[0.354 (0.248–0.505)]	<0.0001	Gender (F versus M)	[0.417 (0.282–0.617)]	<0.0001
Disease duration (years)	[1.004 (0.985–1.023)]	ns	Disease duration	[0.998 (0.976–1.02)]	ns
DAS28 (high versus moderate versus low)	[0.622 (0.542–0.713)]	<0.0001	DAS28 (high versus moderate versus low)	[0.724 (0.626–0.837)]	<0.0001
DMARDs (no versus yes)	[1.248 (0.724–2.151)]	ns	DMARDs (no versus yes)	[0.85 (0.453–1.596)]	ns
Steroids (no versus yes)	[2.109 (1.445–3.078)]	0.0001	Steroids (no versus yes)	[1.775 (1.153–2.734)]	ns
Adalimumab versus infliximab	[1.495 (1.045–2.138)]	ns	Adalimumab versus infliximab	[1.547 (1.041–2.297)]	0.0307
Etanercept versus infliximab	[1.048 (0.69–1.59)]	ns	Etanercept versus infliximab	[0.871 (0.538–1.41)]	ns

HAQ < 0.5 (5th year)	Univariate analysis		HAQ < 0.5 (5th year)	Univariate analysis	
	OR (95% CI)	*P*		OR (95% CI)	*P*

Age (≥65 versus <65 years)	[0.978 (0.964–0.991)]	0.0014	Age (≥65 versus <65 years)	[0.982 (0.967–0.996)]	0.0142
Gender (F versus M)	[0.474 (0.31–0.726)]	0.0006	Gender (F versus M)	[0.571 (0.362–0.901)]	0.016
Disease duration	[1.002 (0.979–1.025)]	ns	Disease duration	[0.995 (0.97–1.022)]	ns
DAS28 (high versus moderate versus low)	[0.729 (0.624–0.853)]	0.0001	DAS28 (high versus moderate versus low)	[0.79 (0.668–0.936)]	0.0063
DMARDs (no versus yes)	[0.702 (0.322–1.53)]	ns	DMARDs (no versus yes)	[0.627 (0.255–1.54)]	ns
Steroids (no versus yes)	[1.851 (1.182–2.898)]	0.0071	Steroids (no versus yes)	[1.832 (1.102–3.045)]	0.0195
Adalimumab versus Infliximab	[0.607 (0.383–0.962)]	ns	Adalimumab versus infliximab	[0.554 (0.337–0.911)]	0.0199
Etanercept versus infliximab	[0.607 (0.364–1.011)]	ns	Etanercept versus infliximab	[0.502 (0.284–0.888)]	0.0179

OR: odds ratio; CI: confidence interval; F: female; M: male; DMARDs: disease modifying antirheumatic drugs; DAS28: 28-joint disease activity score; HAQ: Health Assessment Questionnaire.
